# Perceived human and material costs of disasters as drivers of donations

**DOI:** 10.1111/jasp.12751

**Published:** 2021-02-22

**Authors:** Hanna Zagefka

**Affiliations:** ^1^ Department of Psychology Royal Holloway University of London Egham UK

## Abstract

Are disaster relief appeals more successful if they emphasize the material cost of disaster events in terms of economic damages and need for shelter, food, and health care, or if they emphasize the human cost in terms of psychological suffering and trauma caused? Although giving patterns seem to suggest that large‐scale events that cause widespread material damage (e.g., the Asian Tsunami of 2004) are more successful at eliciting donations than smaller scaled events, it is argued that this pattern is explained by the fact that large perceived material damage leads to more perceived human suffering. In other words, it is the perceived human suffering which is the proximal driver of donations, rather than the material damage itself. Therefore, relief appeals that emphasize the human cost of events are more successful at eliciting donations than appeals that emphasize the material cost of events. This was demonstrated in a study focusing on donations by British participants (*N* = 200) to the Syrian refugee crisis in 2020, a study focusing on donations by British participants to victims of severe weather events in Eastern and Southern Africa in 2020 (*N* = 210), and a study among British participants focusing on a fictitious event (*N* = 150).

## 1 INTRODUCTION

Large‐scale disaster events are becoming more frequent (https://theconversation.com/are‐catastrophic‐disasters‐striking‐more‐often‐83599). At any given time, there is a wealth of different charitable appeals to help victims of a diverse range of adverse circumstances. Given that the frequency of disasters is on the increase, the question of what drives donations to disaster victims is more pressing than ever. What motivates potential donors to open their wallets to assist others in need, often in far‐away places? In this contribution, we will compare the effects of the perceived human and the perceived material cost of disasters. This can answer the question of whether donations can be encouraged more effectively by emphasizing human suffering, or by emphasizing the material damage and resultant need for help.

It is perhaps surprising that the effects of the perceived human and material costs of an event have not directly been studied in comparison to each other. From a marketing point of view, this is an important consideration. Disaster relief appeals must be “snappy” and to the point if they want to not overstretch the readers’ attention span. They cannot afford to present too much detail in setting out the problem. Given the need for brevity, which precludes presentations of multiple dimensions of a problem, fundraisers responding to disaster events need to decide whether to emphasize the human cost of an event, in terms of suffering and trauma, or whether to emphasize the material cost of an event, in terms of material damage, and shortages in the supply of food, shelter, and medication.

Large‐scale events that cause widespread material destruction often trigger an outpouring of donations. One example is the trend observed after the severe 2010 flooding in China which caused in excess of 1,500 deaths and caused over US$20 billion in damage (https://www.cafonline.org/docs/default‐source/about‐us‐publications/caf_wgi2014_report_1555awebfinal.pdf). Another pertinent example is the outpouring of public generosity following the Asian Tsunami of 2004, which caused widespread material damage in several countries.

If disasters that cause severe material damage tend to elicit more donations, this might lead one to propose that donors rationally calculate how much material damage was caused by a given event, how much funds are needed to make good the damage, and then, come to the aid of the victims accordingly. A rational, needs‐based calculation would indeed suggest that the repair of greater material damage requires greater funds, and greater material damage of an event might thereby encourage donations.

However, a wealth of psychological evidence suggests that affective processes more strongly influence helping decisions than cognitive processes and rational considerations (see e.g., Farley & Stasson, 2003; Small, 2011). For example, decisions to help are driven more by information that trigger emotional responses, such as images depicting suffering, than rational considerations about the objective effects and efficiency of a donation (Bergh & Reinstein, 2020). The fact that donation decisions are often not driven by rational considerations is vividly illustrated by results found by Evangelidis and Van den Bergh (2013), who show that the number of fatalities, but not the number of survivors, drive donations to disaster relief appeals. Because dead people clearly cannot benefit from donations, this suggests that rather irrational affective factors drive responses. Several others support the idea that affective impulses are the stronger driver of helping decisions. For example, Bloom (2017) argues that helping is so strongly driven by affective impulses, and that those are so often misguided and are at odds with what rationale decision makers would deem sensible, that he recommends the implementation of countermeasures. Research that shows that affective reactions imputed into the victims of disaster events strongly affect helping responses can also be read as underlining the importance of affect in the context of help for disaster victims (Andrighetto et al., 2014; Cuddy et al., 2007).

Evidence for the importance of affective forces in driving donations is supplemented with evidence that shows that rationale considerations about the effectiveness of a donation do *not* strongly affect donation decisions (e.g., Berman et al., 2018). Although rational, cognitive considerations of the perceived need for help, the potential impact of a donation and so on are not irrelevant (Erlandsson et al., 2015; Zagefka et al., 2012), overall the evidence suggests that their impact will be less strong than that of affective factors.

What emerges, then, is the observation, on the one hand, that events that cause large‐scale damage seem to be better at eliciting donations, and on the other hand, the observation that psychological research suggests that donations are driven by affective factors rather than rational cost‐benefit calculations. Taken together, this suggests that the effect of perceived material cost on donation proclivity might be mediated by perceived human cost in terms of trauma and suffering: events that cause more material damage are perceived to cause more human suffering, and it is the perceived human suffering that proximally drives donations. If perceived human cost is a more direct precursor of donations, this also implies that fundraising campaigns that emphasize the human rather than material cost of an event will be more successful at eliciting donations.

To put the present focus into context, perceived human and material costs of an event are only two features of disaster events that potentially impact on donation decisions. Other features, such as perceptions of what caused the event (Betancourt, 1990; Zagefka et al., 2011) also have important consequences, as do other factors such as the context in which a donation is elicited (Guéguen & Stefan, 2013; Hsee et al., 2013). Features of both the donor (e.g., Erlandsson et al., 2018; Manesi et al., 2019; Wiepking & Bekkers, 2012) and the potential recipient of a donation (e.g., Chapman et al., 2020; Siem et al., 2014) also impact on helping decisions (for an overview, see Zagefka & James, 2015). Features of the donor and the potential recipient might even interact with each other in their effect on donations (James & Zagefka, 2017; Zagefka, 2019). In one extensive review of the literature, Bekkers and Wiepking (2011a) identify eight mechanisms as the most important forces that drive charitable giving: awareness of need; solicitation; costs and benefits; altruism; reputation; psychological benefits; values; and efficacy. The present paper aims to add to this vibrant research field, by delving deeper into the potential effects of two features of disaster events which are quite easily manipulated in fundraising appeals, and which are therefore of especially high applied importance: the perceived material and human costs of events.

### 1.1 Overview of present research

Taken together, it is hypothesized that the perceived human cost of an event is a more proximal, and therefore, more powerful predictor of donations than perceived material cost, and that therefore donation appeals that emphasize the human cost of events will be better at eliciting donations than appeals that merely emphasize the material cost. This was tested in three studies, one asking British participants about their donation intentions to help victims of the Syrian refugee crisis in 2020, and one asking British participants about their donations intentions to help victims of severe weather events in Eastern and Southern Africa in 2020, and one focussing on responses to a fictious event.

## 2 STUDY 1

### 2.1 Method

Study 1 was preregistered prior to data collection (see OSF link below). However, the study that was run differed from the preregistration in one important aspect. The original preregistration was focused on responses to victims of the coronavirus. However, as the media coverage of that particular crisis intensified (in the spring of 2020), we feared ceiling effects on our measures. Therefore, the scenario was changed to the Syrian refugee crisis. Also to avoid ceiling effects, the scales to measure human and material costs were extended to 9‐point scales. Despite these details, the psychological processes, sample size, etc. tested in study 1 were identical to those specified in the preregistration.

#### 2.1.1 Participants

Two hundred British nationals were recruited via the Prolific online platform (*m* = 57, *f* = 142, missing data = 1, mean age = 33.02). A minimum sample of *N* = 150 was determined by a priori power analysis with G*Power for *t* tests (one‐tailed) assuming a small effect size of .02, an alpha level of .05, and requesting a power of .80. To be conservative, we slightly oversampled. In line with Prolific policy, participants in all studies were paid a minimum of £5 per hour (so, around 10 pence for this very short study).

#### 2.1.2 Procedure and measures

*Perceived human cost* was measured with three items: “In your opinion, what is the scale of the Syrian refugee crisis in terms of”… “human suffering,” “human trauma,” and “irreparable mental scarring” (1 = *very small* to 9 = *very big*; *α* = .95).

*Perceived material cost* was measured with three items: “In your opinion, what is the scale of the Syrian refugee crisis in terms of”… “financial loss and damages,” “economic impact,” and “people's livelihoods” (1 = *very small* to 9 = *very big*; *α* = .91). The order in which human and material cost items were presented was randomized between participants.

*Willingness to donate* was measured by telling participants: “There are many charities which are appealing for funds to help Syrian refugees. How do you feel about donating money to such causes?” This was followed with four items (1 = *not at all* to 7 = *very much*, *α* = .94): “I would be willing to donate money,” “I think it is important to donate money,” “I think donating money is the right thing to do,” and “I think everyone should donate money.”

### 2.2 Results and discussion

Bivariate correlations and means for the measures are displayed in Table 1. To test the effects of perceived human and material costs on willingness to donate, a regression was run. Results are displayed in Table 2. As can be seen in the table—and in line with the preregistered hypothesis—perceived human cost of the event was a more important predictor of willingness to donate: perceived human cost significantly predicted the outcome, whereas perceived material cost was not.

**TABLE 1:** Bivariate correlations and means

	1. Donation intentions	2. Perceived human cost	3. Perceived material cost
1. Donation intentions		.43 *** (.47 ***)	.22 *** (.41 ***)
2. Perceived human cost	.46 ***		.45*** (.74 ***)
3. Perceived material cost	.32 ***	.53 ***	
Study 1			
Means	4.72	7.74	7.00
Standard deviation	1.54	1.50	1.70
Scale range	1–7	1–9	1–9
Study 2			
Means	3.88	4.25	4.33
Standard deviation	.94	.66	.65
Scale range	1–5	1–5	1–5
Study 3			
Means	3.41	4.15	4.28
Standard deviation	.91	.76	.69
Scale range	1–5	1–5	1–5

Note

Correlations for study 1 given above the diagonal, correlations for study 2 given below the diagonal, correlations for study 3 given above the diagonal in parentheses.

* *p* < .05;

** *p* < .01;

*** *p* < .001.

**TABLE 2:** Predicting willingness to donate from perceived human and material costs of disaster

	*β*	B	CI_lower_	CI_upper_
**Study 1**				
Overall R^2^= .18 ***				
Human cost	.42 ***	.43	.26	.60
Material cost	.04 ns	.03	−.10	.18
**Study 2**				
Overall R^2^= .22 ***				
Human cost	.39 ***	.56	.30	.75
Material cost	.11 ns	.16	−.10	.41
**Study 3**				
Overall R^2^= .23 ***				
Human cost	.35 ***	.42	.19	.64
Material cost	.16 ns	.21	−.04	.47

* *p* < .05;

** *p* < .01;

*** *p* < .001.

**FIGURE 1:**
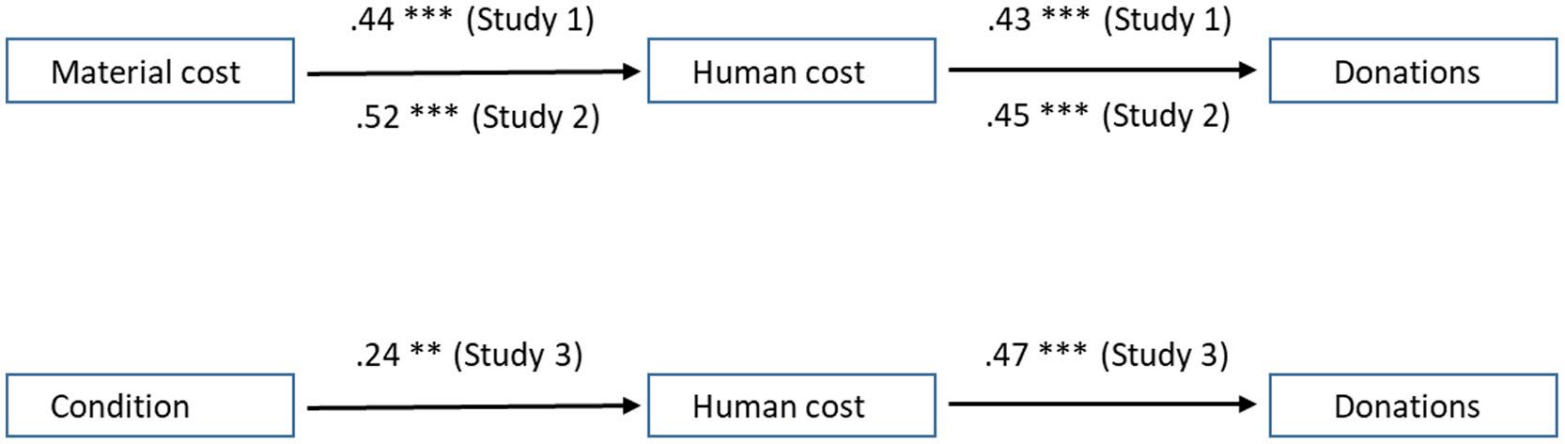
Standardized path coefficients for Studies 1, 2, and 3. ** denotes *p* < .01; *** denotes *p* < .001

In addition, a structural equation model (SEM) was built with AMOS to test the idea that this pattern would emerge because human cost is a more proximal predictor of donations, mediating the effect of perceived material cost. The model fitted the data well, *χ* ^2^ = 0.27, *p* = ns, CFI =.99, RMSEA =.01. Path coefficients are displayed in Figure 1. Importantly, the indirect effect via human costs was significant, standardized indirect effect =.19, *p* <.01, CI_lower_ =.14, CI_higher_ =.28. When switching the order of the independent variable and the mediator, the model did not fit well, *χ* ^2^ = 31.53, *p* < .001, CFI = .63, RMSEA = .39, further confirming that the more proximal predictor of donations is indeed perceived human cost.

## 3 STUDY 2

A further study was conducted to replicate these results in another context, based on real‐life events by focusing on suffering triggered by severe weather in East and South Africa in 2020 (see e.g., https://www.dec.org.uk/appeal/east‐africa‐crisis‐appeal). Again, it was expected that the perceived human cost of an event is a more proximal, and therefore, more powerful predictor of donations than perceived material cost, and that first this would result in human but not material costs emerging as a significant predictor when entered simultaneously, and that second an SEM would support the causal direction from material costs to donations via human cost.

A further aim of this study was to find causal evidence for the preponderance of human costs as a predictor, by experimentally manipulating the messaging of a donations appeal to emphasize the material versus human dimension, and to test whether an appeal that emphasizes the human cost of an event would be better at eliciting donations than an appeal that merely emphasizes the material cost.

### 3.1 Method

#### 3.1.1 Participants

Two hundred ten British nationals were recruited via the Prolific online platform (*m* = 60, *f* = 149, differently identified = 1, mean age = 33.43). The aim was to recruit in excess of *N* = 150, and to conservatively slightly oversample in line with the procedure of the previous study.

#### 3.1.2 Procedure and measures

Participants were told about a real‐life event for which several charities were fundraising at the time, that is, the crisis in East and South Africa caused by severe weather. They were given a short description of the event that emphasized either the material costs, human costs, or both, shown a picture of a woman standing in water in front of a house that was clearly flooded, and asked to take a few seconds to put themselves in the victims’ shoes.

*Perceived human cost* was measured with the same three items as for study 1 (1 = *very small* to 5 = *very large*; *α* = .81).

*Perceived material cost* was measured with the same three items as for study 1 (1 = *very small* to 5 = *very large*; *α* = .82).

*Willingness to donate* was measured by the first three items used in study 1 (1 = *not at all* to 5 = *very much*, *α* = .89).

### 3.2 Results and discussion

Bivariate correlations and means for the measures are displayed in Table 1. The manipulation did not have any significant effects on material costs, *F* (2, 207) = 2.33, *ns*, on human costs, *F* (2, 207) = 1.16, *ns*, or on donations, *F* (2, 207) = 0.20, *ns*. Therefore, because unfortunately the manipulation did not work, the study did not find any causal effect of perceived human costs on donation outcomes.

Despite the failed manipulation, it was possible to analyze the data in correlational terms, and in particular to test whether the results of study 1 would be replicated. A multiple regression was run, predicting willingness to donate from both perceived human cost and perceived material cost of the event simultaneously. Results are displayed in Table 2. As is evident, once again human costs were much more strongly predictive of willingness to donate than material costs.

Again, an SEM was built to test the possibility that the perceived material costs of an event would impact on donation willingness indirectly via perceived human costs. The model fitted the data well, *χ* ^2^ = 2.24, *p* = ns, CFI = .99, RMSEA =.07. Path coefficients are displayed in Figure 1. Importantly, the indirect effect via human costs was significant, standardized indirect effect = .24, *p* < .01, CI_lower_ = .15, CI_higher_ = .31. When switching the order of the independent variable and the mediator, the model did not fit well, *χ* ^2^ = 28.48, *p* < .001, CFI = .76, RMSEA = .36, suggesting that the more proximal predictor of donations is indeed perceived human cost.

## 4 STUDY 3

A further study was conducted, again with the goals of replicating the regression and SEM patterns from the previous studies, and also with the goal of manipulating human costs, to demonstrate a causal effect on willingness to donate. The study was preregistered prior to data collection (see OSF link above).

### 4.1 Method

#### 4.1.1 Participants

One hundred fifty British nationals were recruited via the Prolific online platform, in line with the power analysis cited for previous studies that recommended at least *N* = 150 to achieve a power of .80. Participants were screened to achieve an exact 50/50 male‐female split (mean age = 34.71).

#### 4.1.2 Procedure and measures

Participants were told about a fictitious event (presented as real) and that there was a need for donations because of severe weather which had caused brutal damage, leaving people in need of housing, food, and health care. Participants were randomly allocated to one of two conditions. In one condition, the scenario was only described in terms that emphasized the material cost (“The material damage is huge. Buildings, roads, and infrastructure have been destroyed”), and in the other condition the scenario emphasized the human cost (“The human suffering caused by the event is huge people are suffering, traumatized, and mentally scarred”). To reinforce the message, the scenario also featured a photo of two children sitting next to a building that had obviously been destroyed. In the “human cost salient” condition, the whole picture was displayed. In the “human cost not salient” condition, only the part of the picture featuring the material destruction (but not the children) was displayed.

Participants where then asked whether they would donate to the victims (1 = strongly disagree to 5 = strongly agree), by responding to these three items: “I would donate to the victims,” “I would be motivated to donate to the victims,” and “I think donating to the victims is important,” *α* = .87. They then also indicated whether they would be willing to donate by ticking either “yes” or “no.”

*Perceived human cost* (*α* = .90) and *perceived material cost* (*α* = .82) were then measured with three items each, very similar to those in the previous studies (1 = *very small* to 5 = *very big*).

### 4.2 Results and discussion

Bivariate correlations and means for the measures are displayed in Table 1. To test whether the experimental manipulation was effective in manipulating perceived human cost, an *F* test was run with the “Condition” as independent variable and the manipulation check measure for “human cost” as dependent variables. This yielded that the manipulation significantly shifted perceptions of human costs (*F*(1, 148) = 9.11, *p* < .01, M_humancondition_ = 4.32; *M*_materialconditon_ = 3.96).

To test the preregistered effects on donations, first an *F* test was run with “Condition” as independent variable and the interval scaled donation measure as dependent variable. No significant effect emerged (*F*(1, 148) = .35, *ns*). Moreover, a crosstab analysis looking at the frequency count of “yes” versus “no” donation responses across conditions in a 2*2 table also did not yield a significant effect, *χ* ^2^ = .98, *ns*.

Next, a regression was run replicating the results conducted for the previous studies. Results are displayed in Table 2. As before, and replicating the results of previous studies once again, human costs were much more strongly predictive of willingness to donate than material costs.

Moreover, again an SEM was built. This tested whether the manipulation impacted on donation willingness indirectly via the manipulation check measure of perceived human costs. The model fitted the data well, *χ* ^2^ = 0.82, *p* = ns, CFI = .99, RMSEA = .01. Path coefficients are displayed in Figure 1. Importantly, the indirect effect via human costs was significant, standardized indirect effect = .11, *p* < .01, CI_lower_ = .05, CI_higher_ = .19. Thus, although no direct causal effect of the manipulation on donations emerged in the *F* test, the SEM confirmed that the manipulation (differentially emphasizing human vs. material costs in the description of the event) had an indirect effect on donations, mediated via perceived human costs.

## 5 General discussion

Results supported the prediction that the perceived human cost of a disaster event more strongly drives donation decisions than the perceived material cost. The reason ‐a perceived large scale in terms of material damage increases donation proclivity is that it increases the imputed perceived human suffering. In other words, human costs of a given disaster mediate the effects of material costs on helping outcomes.

This pattern emerged correlationally across all three studies in the regression analyses and the SEM analyses. Although study 2 failed at manipulating perceived human costs effectively, study 3 did manage to manipulate this variable and demonstrated indirect causal effect of the manipulation on donations via perceived human costs which was in line with the hypothesis, although even stronger evidence would have been provided if the manipulation had also affected donation proclivity directly.

One possible explanation for the failure to manipulate perceived human cost (in study 2) and to demonstrate a direct effect of the manipulation on donations (study 3) could be that it is difficult to make disaster scenarios sufficiently engaging for participants of online recruitment platforms, who are often quite routine at completing studies. Although data generated with platforms such as Prolific is generally regarded as being of high quality, participants can be assumed to care less, and be less engaged, than people who actually seek out information on disasters with the intention to donate (e.g., people who browse the websites of disaster relief charities). It is possible that the same manipulations would have yielded stronger effects if the study had not been run via these online platforms.

Having said this, taken together the three studies still yield consistent evidence for the hypotheses, and the results are therefore important in practical terms. The present results demonstrate that material costs of disaster events will often be correlated with human costs, and that in fact the latter mediate the effects of the former on donation intentions. Still, given the need for brevity in disaster relief appeals, the question of which aspect of an event to emphasize in fundraising efforts remains with undiminished importance. Fundraisers need to decide which basic information to convey about an event when asking for contributions. Message framing can strongly affect whether or not donations to disaster victims will be forthcoming (Chapman & Lickel, 2016), and the present results suggest that it will be advantageous to emphasize psychological over material damages caused by a disaster event. For example, rather than describing a disaster as having caused large‐scale material damage to buildings, roads, and infrastructure, it will be more productive to emphasize the effects of the event in terms of human suffering, trauma, and mental scarring. Illustrative images of the human impact also help, of course, as has been demonstrated elsewhere.

Of course, the present research is not without limitations. One obvious restriction is that we only studied donation intentions, not actual donations. There is actually good evidence that self‐reported donation intentions strongly correlate with actual donation behavior. For example, Bekkers and Wiepking (2011b) show that although mean levels of self‐reports are higher than mean levels of actual donations, the two are very highly correlated. Thus, in research such as this one, which is interested in associations between variables rather than an analysis of mean levels, self‐reports can be assumed to be a reasonable proxy for actual donation behavior. Nonetheless, it would of course have been preferable to assess actual donation behavior, and this is a limitation to the current work. Future work could address this, and also—as already indicated above—seek to study these effects in populations other than those reachable through online recruitment platforms.

The present findings confirm the view of scholars who emphasize the importance of emotional rather than rational drivers of helping decisions. For example, there is convincing evidence that the presentation of a single victim leads to more helping than the presentation of groups of victims, because a single victim is better able to elicit emotional responses in potential donors (Kogut & Ritov, 2005; but see Kogut et al., 2015, for some cultural boundary conditions to this). There is also good evidence that people are biased rather than rational and “fair” in their helping decisions, and that they empathize more with in‐group member than out‐group members (Bloom, 2017; Levine et al., 2005; van Leeuwen & Täuber, 2012). Such studies highlight the importance of emotion over cognition in driving helping decisions. The contribution of the present paper was to test this in the context of donations to disaster victims, and to translate this general psychological mechanism into actionable recommendations for fundraisers. On the basis of the present results, then, the recommendation is to emphasize psychological heartache over material damage. Donations will not be motivated most strongly by information about how much help is needed, but by the emotions triggered in the potential donor.

## Data Availability

Data is available on OSF, please see https://osf.io/p6e7u/?view_only=fe412cfbacd1425b99aa9955fb7e5525. Provided are data, syntax, and materials for the three studies (materials are part of the preregistration for studies 1 and 3, and uploaded separately for study 2). The preregistration to study 1 is the document named “Pre‐reg human vs economic impact”. The preregistration to study 3 is the document named “Prereg & Q form F33”.
